# Growth Effects of Some Platinum(II) Complexes with Sulfur-Containing Carrier Ligands on MCF7 Human Breast Cancer Cell Line upon Simultaneous Administration with Taxol

**DOI:** 10.1155/MBD.2002.33

**Published:** 2002

**Authors:** Gordana Bogdanović, Vesna Kojić, Tatjana Srdić, Dimitar Jakimov, Miloš I. Djuran, Živadin D. Bugarčić, Mirjana Baltić, Vladimir V. Baltić

**Affiliations:** 1 lnstitute of Oncology Sremska Kamenica, Novi Sad, lnstitutski put 4 Sremska Kamenica 21204 Serbia and Montenegro; 2 Department of Chemistry University of Kragujevac Faculty of Science R. Domanovića 12, P. O. Box 60 Kragujeva 34000 Serbia and Montenegro

## Abstract

The platinum (II)complexes, *cis*-[PtCl_2_(CH_3_SCH_2_CH_2_SCH_3_)] (Pt1), *cis*-[PtCl_2_(dmso)_2_] (dmso is dimethylsulfoxide; Pt2) and *cis*-[PtCl_2_(NH_3_)_2_] (cisplatin), and taxol (T) have been tested at different equimolar concentrations. Cells were exposed to complexes for 2 h and left to recover in fresh medium for 24, 48 or 72 h. Growth inhibition was measured by tetrazolium WST1 assay Analyses of the cell cycle, and apoptosis were performed by flow cytometry, at the same exposure times. The IC50 value of each platinum(II) complex as well as combination index (CI; platinum(II) complex + taxol) for various cytotoxicity levels were determined by median effects analysis.

MCF7 cells were found to be sensitive to both Pt1 and Pt2 complexe These cisplatin analogues influenced the cell growth more effectively as compared to cisplatin. Cytotoxic effect was concentration and time-dependent. Profound growth inhibitory effect was observed for Pt1 complex, across all its concentrations at all recovery periods. A plateau effect was achieved three days after treatment at Pt1 concentrations ≤ 1 μM. Pt2, however, decreased MCF7 cells survival only for the first 24 h ranging between 50-55%. Pt2 cytotoxicity sharply decreased thereafter, approaching 2 h - treatment cytotoxicity level. The median IC50 values for Pt1 and Pt2 were similar (0.337 and 0.3051 μM, respectively) but only for the first 24 h. The IC50 values for Pt1 strongly depend on the recovery period. On simultaneos exposure of cells to taxol and platinum(II) complexes no consistent effect was found. The Cls for combinations of taxol with Pt1 or Pt2 revealed cytotoxic effects that were in most Cases synergistic (Pt1) or less than addtiive (Pt2). Flow cytometry analysis has shown that each platinum(II) complex induced apoptosis in MCF7 cells. The level of apoptosis correlated with cytotoxicity level for the range concentrations. Both cisplatin analogues, at IC50 concentrations, increased the number of MCF7 cells in G0G1 phase of cell cycle. Pt2-treated cells remained arrested in G0G1 phase up to 72 h after treatment. Combination of Pt2 and taxol caused further arrest of cells in G0G1 phase (24 h) in parallel with strong decrement of G2M phase cells.


### Growth Effects of Some Platinum(Ii) Complexes with Sulfur-Containing Carrier Ligands on MCF7 Human Breast Cancer Cell Line upon Simultaneous Administration with Taxol

Gordana Bogdanović*[1], Vesna Kojić[1], Tatjana Srdić[1], Dimitar Jakimov[1], Miloš I. Djuran[2], Živadin D. Bugarčić[2], Mirjana Baltić[1] and Vladimir V. Baltić[1]

[1]*Institute of Oncology Sremska Kamenica, Novi Sad, Institutski put 4, 21204 Sremska Kamenica, Yugoslavia* < gordanab@ptt.yu >

[2]*Department of Chemistry, University of Kragujevac, Faculty of Science, R. Domanovića 12, P. O. Box 60, 34000 Kragujevac, Yugoslavia*

### ABSTRACT

The platinum (II) complexes, *cis*-[$PtCl_2(CH_3SCH_2CH_2SCH_3)$] (Pt1), *cis*-[$PtCl_2(dmso)_2$] (dmso is dimethylsulfoxide; Pt2) and *cis*-[$PtCl_2(NH_3)_2$] (cisplatin), and taxol (T) have been tested at different equimolar concentrations. Cells were exposed to complexes for 2 h and left to recover in fresh medium for 24, 48 or 72 h. Growth inhibition was measured by tetrazolium WST1 assay. Analyses of the cell cycle and apoptosis were performed by flow cytometry, at the same exposure times. The IC50 value of each platinum(II) complex as well as combination index (CI; platinum(II) complex + taxol) for various cytotoxicity levels were determined by median effects analysis.

MCF7 cells were found to be sensitive to both Pt1 and Pt2 complexes. These cisplatin analogues influenced the cell growth more effectively as compared to cisplatin. Cytotoxic effect was concentration and time-dependent. Profound growth inhibitory effect was observed for Pt1 complex, across all its concentrations at all recovery periods. A plateau effect was achieved three days after treatment at Pt1 concentrations ≤ 1 µM. Pt2 , however, decreased MCF7 cells survival only for the first 24 h ranging between 50-55%. Pt2 cytotoxicity sharply decreased thereafter, approaching 2 h - treatment cytotoxicity level. The median IC50 values for Pt1 and Pt2 were similar (0.337 and 0.3051 µM, respectively) but only for the first 24 h. The IC50 values for Pt1 strongly depend on the recovery period. On simultaneous exposure of cells to taxol and platinum(II) complexes no consistent effect was found. The CIs for combinations of taxol with Pt1 or Pt2 revealed cytotoxic effects that were in most cases synergistic (Pt1) or less than additive (Pt2). Flow cytometry analysis has shown that each platinum(II) complex induced apoptosis in MCF7 cells. The level of apoptosis correlated with cytotoxicity level for the range concentrations. Both cisplatin analogues, at IC50 concentrations, increased the number of MCF7 cells in G0G1 phase of cell cycle. Pt2-treated cells remained arrested in G0G1 phase up to 72 h after treatment. Combination of Pt2 and taxol caused further arrest of cells in G0G1 phase (24 h) in parallel with strong decrement of G2M phase cells.

This study showed that two cisplatin analogues, Pt1 and Pt2, with sulfur-containing carrier ligands, influence the MCF7 cells growth more effectively as compared to the parent drug. They differ in their cytotoxicity profiles and in their interaction with taxol as well. The cell cycle changes and induction of apoptosis in MCF7 cells implicate a programmed cell death pathway in cell-killing.

**Key words:** Platinum complexes; Cell growth; Apoptosis; Cell cycle; Breast cancer cell line.

### INTRODUCTION

Cisplatin, *cis*-[$PtCl_2(NH_3)_2$], is a widely used anticancer drug. The drug's greatest therapeutical impact was found to be on testicular and ovarian cancers /1/. It has also proved to be of benefit in the treatment of wide variety of other solid tumors (head and neck, lung, bladder, colorectal and breast cancer) in combination chemotherapy regimens. Because of its severe toxicity profile and the spontaneous development of drug resistance in tumors, numerous Pt(II) and Pt(IV) complexes have been synthesized and tested for antitumor activity /3/. The principal goal of these investigations is obtaining an antitumor drug with higher solubility, better antitumor activity, and lower toxicity. However, at present, only several platinum(II) complexes (carboplatin and oxaliplatin) have shown substantial anticancer activity in clinical settings. Despite this progress, the search for other platinum chemotherapeutic agents is continuing, since the carboplatin and other second-generation platinum(II) complexes, although less toxic than cisplatin, appear to be highly cross-resistant with cisplatin.

The great majority of the second-generation antitumor active platinum complexes are structural analogues of cisplatin with two ammine or amine groups in *cis*-position. The presence of the N-H groups on the platinum antitumor active complex is likely required for a hydrogen bond donor function, although steric effects cannot be excluded *a priori* /3/. But, most important is that new compounds should lack cross-resistance to cisplatin and carboplatin /4/. It is already known that this requirement can be reached by using non-ammonia ligands.

Cisplatin and taxol are highly suited for combination chemotherapy since they have distinct mechanisms of action /5/. Taxol is used in combination with cisplatin in treatment of metastatic breast cancer patients who have experienced resistance or refractory effects to anthracyclines /1,6/. Taxol, unlike other common antimicrotubule agents, promotes microtubule assembly (enhances the polymerization of tubule) and thus disrupts the dynamic equilibrium within the microtubule system. Due to that, cells are blocked in the late G2/M phase of cell cycle followed by inhibition of cell proliferation.

The aim of the study was to examine the effects of two cisplatin analogues with sulfur-containing carrier ligands on the growth, induction of apoptosis and cell-cycle parameters of MCF7 human breast cancer cell line. The platinum(II) complexes, *cis*-[$PtCl_2(CH_3SCH_2CH_2SCH_3)$] (Pt1) and *cis*-[$PtCl_2(dmso)_2$]) (Pt2), were analyzed as single agents or simultaneously with taxol at equimolar concentrations.

### EXPERIMENTAL

### Chemicals and Materials

Distilled water was demineralized and purified to a resistance greater than 10 MΩ cm. The compounds $K_2[PtCl_4]$, dimethylsulfoxide (dmso) were obtained from Aldrich Chemical Co. and used without further purification. The ligand $CH_3SCH_2CH_2SCH_3$ was prepared by adding two equivalents of $CH_3I$ to the corresponding sodium salt of dithiol ligand in water solution /7/.

All common chemicals were of reagent grade.

### Cell line

MCF7, human breast adenocarcinoma, estrogen receptor positive (ER+) cells grow as monolayer in Dulbecco's modified Eagle's medium (DMEM ) with 4.5% of glucose, supplemented with 10% of fetal calf serum (FCS, NIVNS) and antibiotics: 100 IU/ml of penicillin and 100 μg/mg of streptomycin (ICN Galenika). Cells were cultivated in flasks (Costar, $25cm^2$) at 310 K in the atmosphere of 100% humidity and 5% of $CO_2$ (Heraeus). Exponentially growing viable cells were used through the assays. The viable cells were determined by dye exclusion test (DET) with trypan blue /8/.

### Platinum Complexes

The complexes *cis*-$[PtCl_2(CH_3SCH_2CH_2SCH_3)]$ and *cis*-$[PtCl_2(dmso)_2]$ were prepared from $K_2PtCl_4$ according to literature procedures /9,10/. The purity of these complexes was checked by elemental microanalyses and $^1H$ NMR measurements. The complexes were tested alone or simultaneously with taxol at equimolar concentrations ranging from $10^{-4}$ to $10^{-8}$ M.

### Drugs

Commercially available solution for *i.v.* administration of cisplatin, and taxol (Ebewe, Austria), served as stock solution. The range of drugs concentrations, $10^{-4}$-$10^{-9}$ M, were used, in order to define $IC_{50}$ concentration for particular time point. The substances of adequate concentrations were added in volume of 10 μL/well.

### WST1 assay

Cytotoxicity was evaluated by tetrazolium colorimetric WST1 assay (Boehringer Mannheim). Exponentially growing cells were harvested and plated into 96-well microtitar plates (Costar) at seeding density of $5 \times 10^3$ cells in a volume of 90 μl per well, and preincubated in complete medium at 310 K for 24 hours (h). Tested substances, at twice the required final concentration, in growth medium (10 μl/well) were added to all wells except control. Microplates were incubated for 2 h. After the exposure period medium was changed and cells were left to recover for 24, 48 and 72 h respectively. The wells containing cells without tested substances served as control. Two hours before the end of incubation 10 μl of WST1 solution was added to all wells. Optical density was measured on a spectrophotometer plate reader (Multiscan MCC340, Labsystems) at 492/690 nm. The wells without cells containing complete medium and WST1 only acted as blanks. Inhibition of growth was expressed as a percent of control and cytotoxicity was calculated according to the formula: $(1\text{-}OD_{Test}/OD_{Control}) \times 100$. The substance potency was expressed as the $IC_{50}$ (50% inhibitory concentration).

### Flow Cytometry

*Cell cycle analysis.* Cell suspension ($1 \times 10^6$/ml) was treated with 1ml 0.1% TRITON-X-100 for 5 min at 277 K, followed by 20 μl RNA-ase (1 mg/ml) in PBS, stained with propidium iodide (PI) and analyzed by standard procedure. Flow cytometry was performed on FACS Calibur (Becton Dickinson) flow cytometer.

*Measurement of apoptosis.* Cell suspension ($1 \times 10^6$/ml) was incubated in the dark, in staining buffer containing 20 μL of PI and 20 μL of Annexin-V in HEPES buffer (Annexin-V-FLUOS kit, Boehringer). After the incubation period stained cells were resuspended in HEPES buffer and analyzed by standard procedure. Flow cytometry was performed on a FACS Calibur (Becton Dickinson) flow cytometer.

### Data analysis

The IC50 of platinum(II) complexes and taxol as well as the interaction between them were determined by median effect analysis /11, 12/. The analysis compares the effects of drug combinations to the effects of individual drugs across the entire dose-effect range, indicating if the interaction is synergistic, additive or antagonistic. Data in tables and figures represent the mean of the quadruplicate wells.

### RESULTS

This study evaluates two cisplatin analogues, *cis*-$[PtCl_2(CH_3SCH_2CH_2SCH_3)]$ (Pt1) and *cis*-$[PtCl_2(dmso)_2]$ (Pt2; dmso is dimethylsulfoxide), for their potential to inhibit growth of MCF human

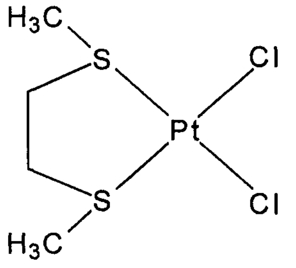

Pt1

*cis*-$[PtCl_2(CH_3SCH_2CH_2SCH_3)]$

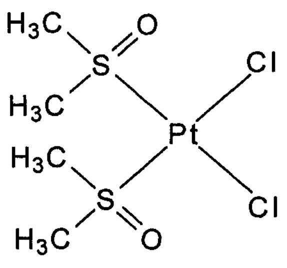

Pt2

*cis*-$[PtCl_2(dmso)_2]$

breast cancer cell line. MCF7 cells were exposed to platinum(II) complexes alone or simultaneously with taxol for 2 hours.The survival of the cells was evaluated by the end of the treatment or after recovery period of 24, 48 and 72 hours respectively. The effects of drug combinations at the IC25, IC50 and IC75 level were determined by median effect analysis. The ability to induce apoptosis and cell cycle changes were analyzed as well.

MCF7 cells were found to be sensitive to both Pt1 and Pt2 complexes (Figure 2). The cytotoxicity profiles of these two platinum(II) complexes are different but each of them influences the cell growth more effectively as compared to cisplatin at equimolar concentrations (Figure 3). Survival rate depends on concentration and recovery time.

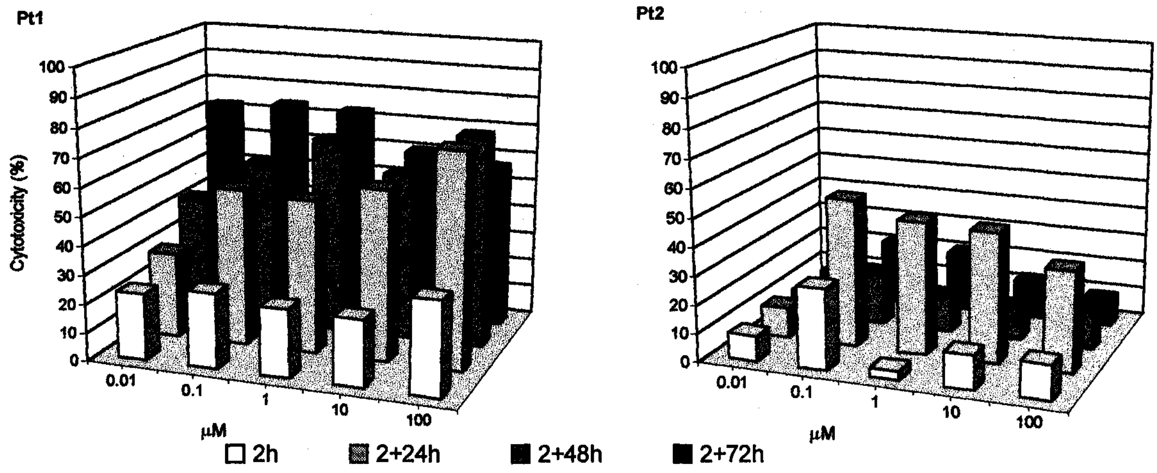

**Fig. 2:** Cytotoxic effect of platinum(II) complexes on MCF7 human breast cancer cell line. Cells ($5x10^3$) were exposed to complexes for 2 h, 24 h after plating. Medium was changed and cells were left to recover for 24, 48 and 72 h respectively. Cytotoxicity was evaluated by tetrazolium WST1 assay at indicated time points. Bars represents mean of quadriplicate wells.

A profound growth inhibitory effect was observed for Pt1 complex, across all its concentrations at each recovery period. Prolonging the recovery time from 24-72 h increased cytotoxicity of Pt1, reaching plateau 72 h post treatment, at concentrations <1 μM. However, Pt2 decreased MCF7 cells survival only for the first 24 h, ranging between 50-55%. The cytotoxicity sharply decreased thereafter, approaching 2 h - treatment cytotoxicity level. Increasing the concentration above 0.1 μM resulted in no additional cytotoxicity resembling Pt1 cytotoxicity pattern. The IC50 values for Pt1 and Pt2 are similar, but only for the first 24 h (0.337 and 0.3051 μM, respectively). The IC50 values for Pt1 decreased with recovery period by the factor 10.

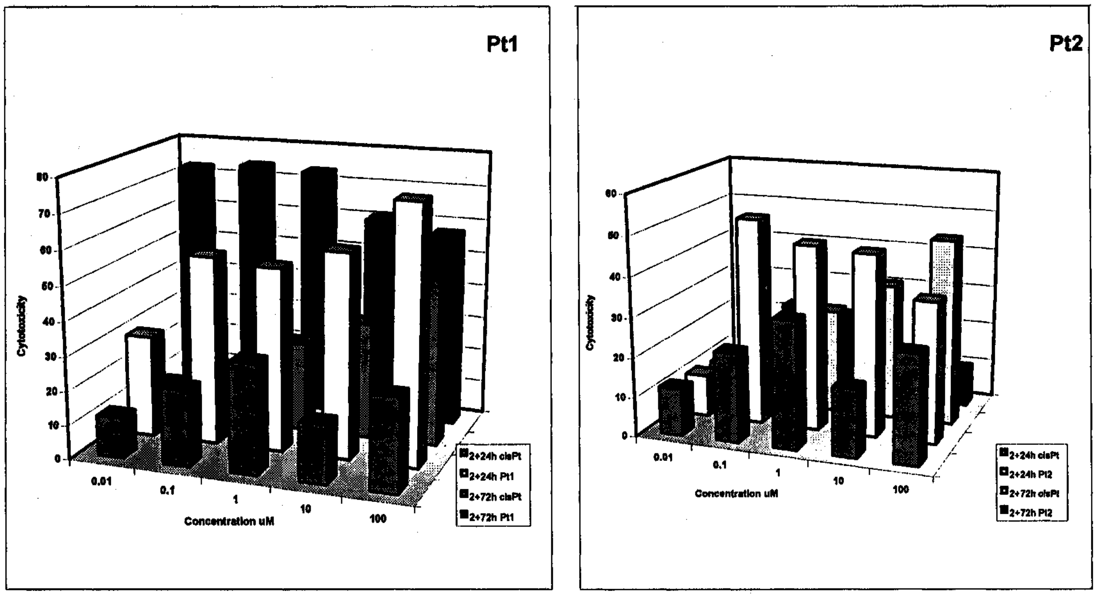

**Fig. 3:** Comparison of growth inhibitory effect of Pt1 and Pt2 complex to cisplatin at 24 h and 72 h post treatment respectively. Maximal growth inhibitory activity of platinum(II) complexes is shown.

The increased survival of MCF7 cells was noticed for both complexes above particular concentrations (>1 μM for Pt1 and >0.1 μM respectively). In order to check out whether the number of surviving cells was really increased or there was only change in the cell metabolic activity, DET assay was performed under the same experimental conditions. The results obtained highly corresponded to those of tetrazolium assay. The linear correlation coefficients were $r_{Pt1} = 0.9361$ and $r_{Pt2} = 0.9086$ respectively (data not shown).

Combinations of platinum(II) complexes and taxol were analyzed by median effect method, primarily under the assumption that drug mechanisms of action were mutually nonexclusive *i.e.* were completely independent. A drug combination index (CI) was calculated for three different levels of cytotoxicity (25; 50 and 75%). On simultaneous exposure of cells to taxol and platinum(II) complexes no consistent effect was found. The CIs for drug combinations revealed cytotoxic effects that were in most cases synergistic (Pt1) and less than additive (Pt2). Median effect analysis showed that interaction of platinum(II) complexes and taxol varies, depending not only on the type of complex, but also on the cytotoxicity level (Figures 4 and 5).

It is well known that cisplatin can induce apoptosis in various cells, in any cell cycle phase, as a function of drug concentration and exposure duration, but a period of cell cycle "stasis" precedes the onset of apoptosis [5, 13, 14]. We examined whether platinum(II) complexes can induce apoptotic cell death in MCF7 cells. Flow cytometry analysis has shown that each platinum(II) complex induced apoptosis in MCF7 cells. Prolonging the exposure time of cells to platinum(II) complexes, at IC50 concentration, from 2 to 24 h, increased the total number of apoptotic cells. Both Pt1 and Pt2 induce higher apoptosis level at lower concentrations during the first 24 h post treatment that correlates with cytotoxicity profile of the complexes (data not shown).

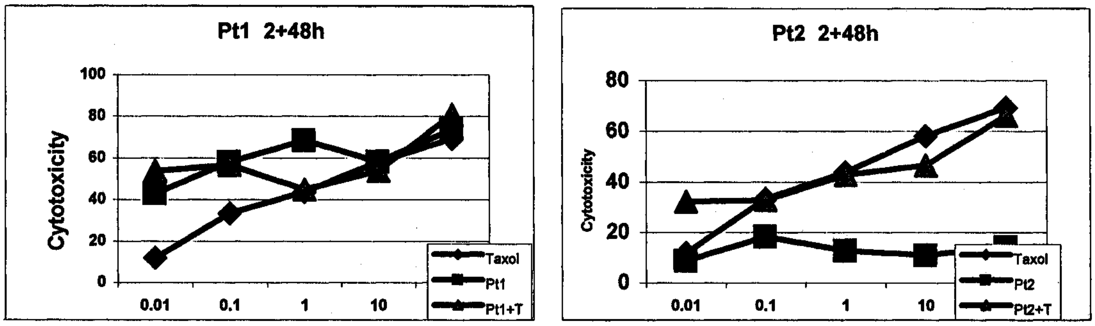

**Fig. 4:** Effects of platinum(II) complexes alone and in combination with taxol on MCF7 cell line survival, determined 48 h post treatment. 24 h after plating cells ($5x10^3$) were exposed to complexes for 2 h. Medium was changed and cells were left to recover. Survival was evaluated by tetrazolium WST1 assay at indicated time points. Bars represents mean of quadruplicate wells.

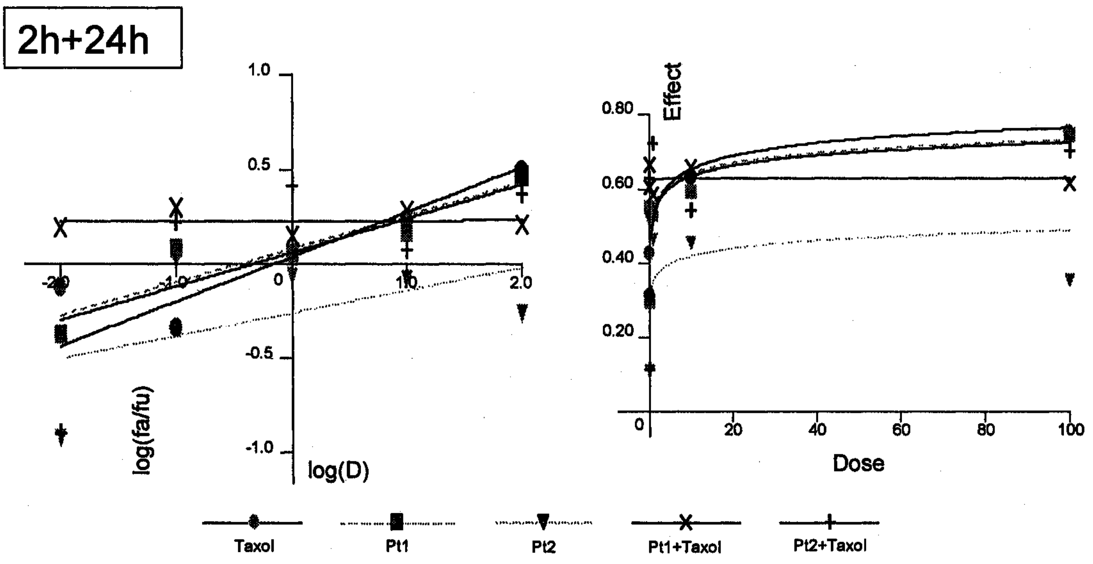

| | ED50 | ED75 | ED90 | Dm | m | r |
|---|---|---|---|---|---|---|
| Taxol | N/A | N/A | N/A | 0.43738 | 0.18069 | 0.89184 |
| Pt1 | N/A | N/A | N/A | 0.33718 | 0.17717 | 0.92431 |
| Pt2 | N/A | N/A | N/A | 133.80355 | 0.12122 | 0.49542 |
| Pt1+Taxol | 8.8644e-104 | 3.3539e+109 | 1.#INF | 1.6878e-104 | 0.00222 | 0.05507 |
| (1:1) | 8.8644e-104 | 2.7985e+218 | 1.#INF | Mutually non-exclusive | | |
| Pt2+Taxol | 1.52038 | 0.35022 | 0.08091 | 0.66282 | 0.23805 | 0.69832 |
| (1:1) | 1.52788 | 0.35024 | 0.08091 | Mutually non-exclusive | | |

**Fig. 5:** IC50 and combination index(CI) values determined by *Median Effect* analysis

Taxol alone induced strong apoptotic response (38.22%), while in combination with Pt2 under the same experimental conditions, total number of apoptotic cells decreased (30.25%). Pt1 induced higher total number of apoptotic cells than Pt2, (24.15 and 13.88 respectively ) at 24 h post treatment but in all other time points the values were similar (Table 1).

Although cisplatin is cycle-phase nonspecific, cells treated with cytotoxic cisplatin concentrations may remain arrested at one or more steps of the cell cycle for up to several days prior to cell death /15/. Both platinum(II) complexes also induced cell cycle perturbations. Cells treated at IC50 concentrations of Pt1 and Pt2 accumulated in G0G1 phase of cell cycle. Pt1 transiently (24 h) increased the number of cells in S phase of cell cycle. Pt2-treated cells remained arrested in G0G1 up to 72 h post exposure. Taxol inhibits cell cycle traverse at the G2M phase junction /16/. It was found that Pt2 in combination with taxol caused further arrest of cells in G0G1 phase (24 h) in parallel with strong decrease of cells in G2M phase cells (Table 2).

**Table 1.**

Time-related effect of platinum(II) complexes on induction of apoptosis in MCF7 cells.

| | APOPTOSIS (%): | | | | | |
|---|---|---|---|---|---|---|
| | **total** (early+late) | | | | | |
| Drugs | **2h** | **4h** | **24h** | **2h+24h** | **2h+48** | **2h+72** |
| **0** | **19.37** (5.73+13.64) | **18.71** (5.48+12.87) | **26.9** (5.79+21.11) | **19.09** (7.41+11.68) | **27.96** (0.63+27.33) | **21.5** (3.76+17.74) |
| ***cis*-Pt** | **23.44** (12.6+10.84) | ND | **ND** | **20.08** (5.68+14.4) | **20.77** (1.13+19.63) | **20.99** (3.25+17.74) |
| **Pt1** | **24.79** (4.38+20.41) | **23.35** (8.79+14.56) | **31.33** (7.11+24.22) | **24.15** (9.57+14.58) | **23.54** (0.64+22.9) | **22.09** (4.89+17.2) |
| **Pt2** | **23.88** (10.36+13.25) | **26.16** (3.7+22.46) | **33.99** (6.41+27.58) | **13.88** (2.85+11.03) | **22.25** (1.13+21.12) | **22.28** (3.25+19.03) |
| **Taxol** | ND | ND | ND | **38.22** (5.12+33.1) | **39.68** (6.98+32.7) | ND |
| **Pt2+T** | ND | ND | ND | **30.25** (3.24+27.01) | ND | ND |

Platinum(II) complexes were applied at IC50 concentrations. The apoptosis was analyzed by Annexin-V FLUOS assay. Data are given as a percent of apoptotic cell number.

**Table 2.**

Effects of platinum(II) complexes on DNA content of MCF7 cells.

| | CELL CYCLE PHASES | | | | | | | | | | | |
|---|---|---|---|---|---|---|---|---|---|---|---|---|
| | G0G1 | | | | S | | | | G2M | | | |
| Drugs | 2h | 2h+24 h | 2h+48 | 2h+72 | 2h | 2h+24 h | 2h+48 | 2h+72 | 2h | 2h+24 h | 2h+48 | 2h+72 |
| 0 | 79.49 | 77.73 | 73.61 | 93.67 | 3.73 | 8.34 | 25.8 | 0.16 | 16.78 | 13.93 | 0.59 | 6.17 |
| *cis*-Pt | 79.80 | 79.14 | 77.55 | 94.39 | 0.97 | 9.90 | 21.84 | 0.89 | 19.22 | 10.96 | 0.61 | 4.72 |
| Pt1 | 79.05 | 68.79 | 82.62 | 92.66 | 0.5 | 0.14 | 17.38 | 3.86 | 20.45 | 31.07 | 0 | 3.48 |
| Pt2 | 76.61 | 80.54 | 99.31 | 91.39 | 12.23 | 4.9 | 0.68 | 0 | 11.16 | 14.05 | 0.01 | 8.61 |
| Pt2+T | ND | 72.55 | ND | ND | ND | 23.87 | ND | ND | ND | 3.58 | ND | ND |

Platinum(II) complexes were applied at IC50 concentrations. The DNA content was analyzed by propidium iodide. Data are given as a percent of cell number at particular cell cycle phase.

### DISCUSSION

In this paper we report on *in vitro* results of antitumor activity of some non-classical platinum(II) complexes against human breast cancer cell line. This study showed that two platinum(II) complexes, Pt1 and Pt2, with sulfur-containing carrier ligands, strongly inhibited growth of MCF7 cells in a dose and time-dependent manner. They also induced apoptosis and cell cycle changes in treated cells. The interaction of platinum(II) complexes and taxol varied depending on the type of complex and the cytotoxicity level.

The tested platinum(II) complexes are cisplatin analogues. It is recognized that manipulation of the structure of the leaving groups appears to influence tissue and intracellular distribution of the complexes, but upon interacting with DNA, the stable, carrier groups presumably determine the structure of the adduct. The ultimate aim of the modifications of the parent drug is to make analogues that produce a different spectrum of DNA lesions and so circumvent the problem of resistance to cisplatin /1/. It seems that differences related to carrier ligands influence both types and frequencies of DNA lesions formed, and consequently, various growths inhibiting activity could be expected.

*Cis*-configuration of the tested platinum(II) complexes was identified as potentially critical for antineoplastic activity. They are platinum(II) structures assuming planar shape. Both of them have chlorine atoms for so-called leaving groups. The "carrier" ligands are cyclic moieties in Pt1, but not in Pt2 complex. It is already known that structural difference of the carrier ligand may greatly alter the spectrum of antitumor activity of platinum(II) complexes. The carrier ligand of Pt1 complex consists of two thioether (-$SCH_3$) groups and its chelation to the Pt center forms a very stable five-member ring. This ring contributes to higher stability of Pt1 than the corresponding Pt2 complex. Difference in the stability between these two complexes can also be correlated with difference in their toxicity. The carrier ligand of Pt2 complex consists of two molecules of dimethylsulfoxide. The lower toxicity of Pt2 might be attributed to its faster detoxification. As data on pharmacokinetics and pharmacodinamics of the Pt1 and Pt2 complexes have not been available so far, we can only speculate on their structure-related activity assuming that, as cisplatin analogues, cytotoxic mechanism(s) similar to that of the parent drug could be expected.

The differences among the complexes were also found when the effects of combination with taxol were studied. Various kinetic of apoptosis-induction and cell cycle changes, induced by individual platinum(II) complex, can explain, in part, resulting differences of drug combinations. Platinum(II) complexes, especially Pt2, induce apoptosis earlier and arrest cells in G0G1 phase of cell cycle. Taxol inhibits cell cycle traverse at the G2M phase junction /16/. By arresting cells in G0G1 phase of cell cycle Pt2 inhibited both taxol-induced mitotic arrest and apoptotic death. So, less than additive cytotoxic effect of Pt2 and taxol combination corresponds to early and strong arrest of cells in G0G1 phase by Pt2.

We want to point out that the interaction of taxol and Pt-complexes was evaluated only on simultaneous exposure of MCF7 cells to the drugs. It is known that interaction of taxol and cisplatin is highly schedule- and cell-dependent /17,18/. Kano *et al.* found that on sequential exposure to paclitaxel first, followed by cisplatin, additive effects were observed in different cell lines including MCF7 cells /17/. On simultaneous exposure to the drugs additive and subadditive effects were obtained in A549, MCF7 and PA1 cells. Our results with Pt1 and Pt2 complexes at least in part correspond with their results. Different mechanisms by which cisplatin may exert dominance over taxol, suggested in some studies /5/, must be kept in mind as well as when analysis of Pt2 and taxol interaction is concerned.

### CONCLUSION

This study showed that two Pt1 and Pt2 complexes containing thio ligands influence the MCF7 cells growth more effectively as compared to the parent drug. However, they differ in their cytotoxicity profiles and in their interaction with taxol as well. The cell cycle changes and induction of apoptosis in MCF7 cells implicate a programmed cell death pathway in cell-killing.

### ACKNOWLEDGMENTS:

This work was funded in part by the Ministry of Science, Technology and Development of the Republic of Serbia (Grant 1254). We are grateful to Mrs. Ljiljana Krmpot for excellent technical assistance.

### REFERENCES

1. J. P. O'Dwyer, W. Johnson and C. Hamillton, Cisplatin and its analogues, In: T. V. De Vita, J. R. S. Hellman and A. S. Rosenberg (Eds), *Cancer principles and practice of oncology,* Philadelphia, Lippincott-Raven, 1997; 375-512.
2. M. J. Bleomink and J. Reedijk, In: *Metals ions in biological systems*, A. Sigel and H. Sigel (Eds), Marcel Dekker, Inc, Basel, 32, 1996; 641-685.

3. E. L. M. Lempers and J. Reedijk, *Adv. Inorg. Chem.*, **37**. 175 (1992).
4. J. Reedijk, *Chem. Comm.*, **1996**, 801.
5. L. P. Judson, M. J. Watson, A. P. Gehrig, C. W. Fowler and J. S. Haskill, *Cancer Res.*, **59**, 2425 (1999).
6. K. Gerald and M. C. Evoy, Paclitaxel, In: K. Gerald and M. C. Evoy (Eds), *Drug Information*, American Hospital Formulary Service, 1997; 841.
7. A. I. Vogel (Ed), *Practical Organic Chemistry*, Longman, London, 1972, p. 498.
8. G. Bogdanović, J. Raletić-Savić and N. Marković, *Arch. of Oncology*, **2**, 181 (1994).
9. M. I. Djuran, S. U. Milinković, A. Habtemariam, S. Parsons and P. J. Sadler, *J. Inorg. Biochem.*, **88**, 268 (2002).
10. J. H. Price, A. N. Williamson, R. F. Schramm and B. B. Wayland, *Inorg. Chem.*, **11**, 1280 (1972).
11. P. R. Perz, K. A. Godwin, M. L. Handel and C. T. Hamilton, *Eur. J. Cancer*, **29A** (3), 395 (1993).
12. T. C. Chou and P. Talalay, *Adv. Enzyme Reg.*, **22**, 27 (1984).
13. R. A. Huddart, J. Titley, D. Robertson, G. T. Williams, A. Horwich and C. S. Cooper, *Eur. J. Cancer*, **31A** (5), 739 (1995).
14. M. Hong, M. D. Lai, Y. S. Lin and M. Z. Lai, *Cancer Res.*, **59**, 2847 (1999).
15. W. Steven, T. S. Schimke, T. R. Schimke, *Apoptosis*, **1994**, 223.
16. S. Y. Sun, P. Yue, G. S. Wu, W. S. El-Deiry, B. Shroot, W. K. Hong, R. Lotan, *Cancer Res.*, **59**, 2829 (1999).
17. Y. Kano, M. Akutsu, S. Tsunoda, K. Suzuki and Y. Yazawa, *Cancer Chemother. Pharmacol.*, **37**, 525 (1996).
18. N. Cordes and L. Plasswilm, *Anticancer*, **18**, 1851 (1998).

